# Treatment Persistence and Adherence with Overactive Bladder Medications in Taiwan: A Retrospective Database Analysis

**DOI:** 10.1016/j.euros.2026.03.020

**Published:** 2026-04-21

**Authors:** Shiau-Han Chen, Chung-Yu Chen, Farid Abdul Hadi

**Affiliations:** a School of Pharmacy, Kaohsiung Medical University, Kaohsiung, Taiwan; b Department of Pharmacy, Kaohsiung Medical University Hospital, Kaohsiung, Taiwan; c Department of Medical Research, Kaohsiung Medical University Hospital, Kaohsiung, Taiwan; d Astellas Pharma Singapore Pte Ltd, Singapore

**Keywords:** Adrenergic beta-3 receptor agonists, Bone fracture, Medication adherence, Medication persistence, Muscarinic antagonists, Urinary bladder, Overactive

## Abstract

**Background:**

Overactive bladder (OAB) is commonly treated with mirabegron or antimuscarinics, but real-world evidence on treatment persistence, adherence, and fall/fracture outcomes in Taiwan remains limited.

**Objective:**

To assess treatment persistence, adherence, and associated outcome rates of falls/fractures, in patients receiving OAB treatment with mirabegron or antimuscarinics in Taiwan.

**Design, setting, and participants:**

Retrospective, longitudinal, observational study of routine clinical practice within the Taiwanese National Health Insurance Research Database. Eligibility: age ≥20 years; ≥1 dispensing for a new OAB index drug (January 1, 2012 to December 31, 2018); continuous enrollment 1 year before and after index prescription date.

**Intervention:**

OAB Treatment with mirabegron or antimuscarinics.

**Outcome measurements and statistical analysis:**

Primary endpoint: treatment persistence (time to discontinuation [TTD] or switch) and adherence (proportion of days covered [PDC]); secondary endpoint: incidence of falls/fractures. Multivariate Cox proportional hazards analyses were applied to estimate adjusted hazard ratios (aHRs) with 95% confidence intervals (CIs), describing associations between treatment groups and outcomes.

**Results and limitations:**

Overall, 1,954,544 patients were eligible (median age 60 years). Median (interquartile range [IQR]) TTD was longer with mirabegron (56 [19–168] days) than antimuscarinics (14 [5–42] days) (risk of discontinuation aHR 1.58 [95% CI 1.55–1.61]; p < 0.001). Median (IQR) PDC was significantly higher with mirabegron (0.18 [0.06–0.52]) than antimuscarinics (0.03 [0.01–0.12]; p < 0.001). Mirabegron and antimuscarinics had no statistically significant difference in the risk of composite falls/fractures (aHR 1.05 [95% CI 0.91–1.20]; p = 0.519), falls (1.33 [0.66–2.68]; p = 0.431), and fractures (1.04 [0.90–1.19]; p = 0.623).

**Conclusions:**

Persistence and adherence were significantly greater with mirabegron than antimuscarinics in patients receiving OAB treatment in Taiwan. No statistically significant association was observed for the incidence of falls/fractures between treatment groups. However, given the wide 95% CIs, the findings should not be interpreted as equivalence.

## Take Home Message

Patients receiving mirabegron had better persistence and adherence than those receiving antimuscarinics in Taiwan, with no significant differences in the risk of falls/fractures between mirabegron and antimuscarinics. These findings provided treatment patterns for overactive bladder pharmacotherapy and may support treatment decisions.

ADVANCING PRACTICE **What does this study add?**

This study provides large-scale real-world evidence from Taiwan on overactive bladder treatment patterns. Mirabegron was associated with better persistence and adherence than antimuscarinics. No statistically significant difference in falls or fractures was observed between treatment groups.

**Clinical Relevance**

Real-world treatment continuation is a key determinant of effectiveness in overactive bladder care. These data suggest that mirabegron may offer an advantage over antimuscarinics for long-term medication use in clinical practice, whereas the relationship between treatment choice and falls/fractures remains inconclusive.

**Patient Summary**

In this study, we looked at how long patients in Taiwan continued treatment for overactive bladder and how regularly they took their medication. We found that patients treated with mirabegron had better treatment persistence and adherence than those treated with antimuscarinics. We did not observe a statistically significant difference in falls or fractures between the two groups.

## 1 Introduction

Overactive bladder (OAB) is defined as urinary urgency, with or without urgency urinary incontinence, usually with increased daytime frequency and nocturia, in the absence of infection or other obvious pathology. OAB is a common condition, affecting approximately 546 million adults aged ≥20 yr worldwide in 2018 [1] and 20.8% of adults aged ≥40 yr in China, Taiwan, and South Korea in 2015 [2]. The prevalence of OAB is similar in men and women [3], [4], and increases with age [2], impacting health care costs and health-related quality of life (HRQoL) [5].

Pharmacotherapy with antimuscarinics and/or β3-adrenoreceptor agonists (mirabegron or vibegron) is recommended by international OAB guidelines [6]. Both classes reduce the occurrence of bothersome urinary incontinence and micturition symptoms [7] with similar efficacy, whereas mirabegron has been reported to have fewer drug-related adverse events (AEs) than antimuscarinics [7], [8], [9]. Real-world studies suggest mirabegron is associated with greater treatment persistence and adherence versus antimuscarinics [10]. The higher rates of treatment discontinuation with antimuscarinics appear to be independent of baseline factors (age, prior treatment) [11], [12] and may be due to a higher rate of anticholinergic AEs (e.g., dry mouth and constipation) [7], [8].

Older individuals (≥65 yr) represent a high proportion of the OAB population and may have multiple comorbidities requiring polypharmacy [2], [13]. A Japanese database study showed that 2%–12% of older individuals receive anticholinergic medication [14], and older patients may be more vulnerable to off-target anticholinergic effects [13], [15]. A higher anticholinergic burden is associated with increased rates of falls/fractures in patients with OAB [15], and these are major causes of morbidity and mortality among older adults [17]. Overall, cumulative anticholinergic effects should be considered when prescribing treatment for OAB [13], [18].

This study assessed treatment persistence and adherence, and associated outcome rates of falls/fractures, in patients receiving treatment for OAB with mirabegron or antimuscarinics in Taiwan.

## 2 Materials and methods

### 2.1 Study design and objectives

This was a retrospective, longitudinal, observational study involving patients receiving treatment for OAB (an antimuscarinic or mirabegron) in Taiwan. Data were collected from the National Health Insurance Research Database (NHIRD), which contains information (reimbursement, ambulatory, inpatient, emergency visit claims, prescription records, and deaths) for approximately 99.9% of Taiwan’s population [19], [20].

The primary objective was to evaluate the pharmacoepidemiology (utilization, persistence, and adherence) of antimuscarinics and mirabegron in adult patients. The secondary objective was to describe the association between the incidence of falls/fractures and these treatments. This observational study was descriptive rather than causal, aiming to characterize associations between treatment choice and the incidence of falls rather than to establish causal effects. This study is reported in accordance with the STROBE guidelines [21] and was approved by the IRB of Kaohsiung Medical University Hospital (KMUHIRB-E(I)-20210151) and conducted in accordance with the Declaration of Helsinki.

### 2.2 Study population

Adult outpatients (≥20 yr) with ≥1 dispensing record for a new OAB target drug (mirabegron, solifenacin, tolterodine, oxybutynin, trospium, or propiverine) between January 1, 2012, and December 31, 2018, were consecutively included (Supplementary Fig. 1). The first prescription date during the selection period was defined as the index date. Patients were required to have continuous enrollment for 1 yr before the index date (to assess eligibility) and 1 yr after the index date (to assess outcomes). Exclusion criteria included pre and postindex period of <1 yr, the same index OAB treatment during the preindex period, diagnosis of stress incontinence (International Classification of Diseases [ICD]-10 N39.3 or equivalent) or mixed incontinence (ICD-10 N39.4 or equivalent), and a prescription for stress incontinence during the preindex period, onabotulinumtoxin A and/or surgical intervention for OAB during the preindex period, and diagnosis of urinary tract infection (ICD-10 N39.0 or equivalent) ≤1 mo before the index date.

### 2.3 Endpoints

The primary endpoints were treatment persistence (time to discontinuation [TTD] or switch during the postindex period) and adherence to the index drug (proportion of days covered [PDC], the sum of days “covered” by medication supply during the postindex period). The index drug was considered discontinued if >30 days had elapsed after the last day of the previous supply without a new prescription, or if there was a switch to another OAB drug. Switching was defined as the prescription of a nonindex OAB drug, and if the index drug was not dispensed continuously, a dose change was not considered switching.

The secondary endpoint was the incidence of falls and/or fractures during the postindex period. Events were defined as concurrent emergency department visits, hospitalizations, surgery, or prescriptions due to falls or fractures, using the corresponding primary or secondary ICD codes.

### 2.4 Statistical analysis

Continuous data were summarized using mean, standard deviation, median, and interquartile range (IQR). Categorical data were summarized by frequencies and percentages. Chi-squared tests were used to analyze categorical variables, A student’s *t* test and analysis of variance were used for continuous variables, and Mann-Whitney U and Kruskal-Wallis H tests were used to analyze non-normally distributed data. A *p* value <0.05 was considered statistically significant.

Persistence analyses were presented using Kaplan-Meier curves. Estimated median TTD with 95% confidence intervals (CIs), and the proportion of patients persistent with the index drug at 12 mo, were also reported. Patients were censored at add-on therapy (second OAB medication or index drug dose escalation) during the follow-up period, or at the end of follow-up without discontinuation. Multivariate analyses using stepwise Cox regression models, which adjusted for baseline characteristics, comorbidities, and concomitant medication, were used to analyze the risk of discontinuation between mirabegron and all antimuscarinics, and results were reported as adjusted hazard ratios (aHRs) with 95% CIs. All comorbidities and concomitant medications were modeled as categorical indicator variables, whereas age and the number of concurrent medications (polypharmacy) were included as continuous baseline covariates. Subgroup investigations assessed by age, sex, and treatment status (experienced, naïve) were conducted. Sensitivity analyses evaluated the impact of changing the period without prescription renewal used to define discontinuation from 30 d after the last day of the previous supply to 15, 60, and 90 d.

Median PDC was used to assess adherence, and cumulative curves of PDC were plotted. For falls/fractures, propensity-score matching (PSM) was used to improve comparability between treatment groups. Falls/fractures data were reported using incidence rates; a Cox proportional hazards model was used to analyze the association between incidences of falls/fractures and the index drugs, reported as aHRs with 95% CIs. Subgroup analysis by age and sex was conducted, and sensitivity analyses evaluated the impact of excluding patients diagnosed with fractures ≤1 yr prior to the index date and using different definitions of fractures and falls based on hospitalization or two/three outpatient records or one record with corresponding x-rays.

All analyses were performed using SAS Software, version 9.4.

## 3 Results

### 3.1 Baseline characteristics

Overall, 8 155 533 patients received a target OAB drug between January 2012 and December 2018, and 1 954 544 patients constituted the study population (Supplementary Fig. 2). Oxybutynin was the most prescribed drug (*n* = 931 590 [47.7%]) followed by trospium (*n* = 388 234 [19.9%]), solifenacin (*n* = 229 086 [11.7%]), tolterodine (*n* = 207 173 [10.6%]), mirabegron (*n* = 102 531 [5.3%]), and propiverine (*n* = 95 930 [4.9%]; Table 1). Median (IQR) age at index date was 60 (46–72) yr in the overall population; mirabegron users (68 [58–77] yr) were significantly older than antimuscarinic users (59 [45–72] yr; *p* < 0.001). Most patients were female (58.5%), including 41.1% in the mirabegron and 59.5% in the antimuscarinic cohort.

**Table 1:** Demographic and clinical characteristics for patients prescribed mirabegron and antimuscarinics (counts and percentages)

Parameter	Mirabegron (*n* = 102 531)	Oxybutynin (*n* = 931 590)	Propiverine (*n* = 95 930)	Solifenacin (*n* = 229 086)	Tolterodine (*n* = 207 173)	Trospium (*n* = 388 234)	All antimuscarinics (*n* = 1 852 013)	Total (*n* = 1 954 544)
Age in years^a^								
Median (IQR)	68 (58–77)	58 (43–71)	62 (49–73)	64 (52–75)	68 (58–77)	61 (47–74)	59 (45–72)	60 (46–72)
Sex, *n* (%)								
Female^a^	42 102 (41.1)	583 786 (62.7)	52 595 (54.8)	119 952 (52.4)	123 433 (59.6)	221 988 (57.2)	1 101 754 (59.5)	1 143 856 (58.5)
Male^a^	60 429 (58.9)	347 804 (37.3)	43 335 (45.2)	109 134 (47.6)	83 740 (40.4)	166 246 (42.8)	750 259 (40.5)	810 688 (41.5)
Geographic region, *n* (%)								
North	49 145 (47.9)	389 973 (41.9)	47 210 (49.2)	117 208 (51.2)	105 350 (50.9)	184 110 (47.4)	843 851 (45.6)	892 996 (45.7)
Central	13 183 (12.9)	226 083 (24.3)	3685 (3.8)	34 817 (15.2)	39 079 (18.9)	83 689 (21.6)	387 353 (20.9)	400 536 (20.5)
South	34 670 (33.8)	294 402 (31.6)	44 423 (46.3)	66 734 (29.1)	59 064 (28.5)	103 124 (26.6)	567 747 (30.7)	602 417 (30.8)
Other	5533 (5.4)	21 132 (2.3)	612 (0.6)	10 327 (4.5)	3680 (1.8)	17 311 (4.5)	53 062 (2.9)	58 595 (3.0)
Treatment status, *n* (%)								
Experienced^a^	22 548 (22.0)	39 168 (4.2)	11 754 (12.3)	40 670 (17.8)	25 999 (12.5)	37 158 (9.6)	154 749 (8.4)	177 297 (9.1)
Naïve^a^	79 983 (78.0)	892 422 (95.8)	84 176 (87.7)	188 416 (82.2)	181 174 (87.5)	351 076 (90.4)	1 697 264 (91.6)	1 777 247 (90.9)
Comorbidities, *n* (%)^a^								
0	53 295 (52.0)	658 969 (70.7)	61 142 (63.7)	136 341 (59.5)	135 752 (65.5)	271 038 (69.8)	1 263 242 (68.2)	1 316 537 (67.4)
1–2	43 891 (42.8)	251 735 (27.0)	31 756 (33.1)	84 608 (36.9)	65 626 (31.7)	107 568 (27.7)	541 293 (29.2)	585 184 (29.9)
3–5	5291 (5.2)	20 729 (2.2)	3008 (3.1)	8085 (3.5)	5755 (2.8)	9543 (2.5)	47 120 (2.5)	52 411 (2.7)
>5	54 (0.1)	157 (<0.1)	24 (<0.1)	52 (<0.1)	40 (<0.1)	85 (<0.1)	358 (<0.1)	412 (<0.1)
CCI^a^								
Mean (SD)	1.89 (2.03)	1.18 (1.72)	1.43 (1.85)	1.63 (1.96)	1.33 (1.78)	1.23 (1.74)	1.28 (1.77)	1.31 (1.79)
Concomitant medication, *n* (%)								
0^a^	33 443 (32.6)	570 590 (61.2)	49 171 (51.3)	100 230 (43.8)	110 559 (53.4)	236 219 (60.8)	1 066 769 (57.6)	1 100 212 (56.3)
1–2^a^	65 658 (64.0)	346 741 (37.2)	44 880 (46.8)	122 218 (53.4)	91 989 (44.4)	144 867 (37.3)	750 695 (40.5)	816 353 (41.8)
3–6^a^	3430 (3.3)	14 259 (1.5)	1879 (2.0)	6638 (2.9)	4625 (2.2)	7148 (1.8)	34 549 (1.9)	37 979 (1.9)
Index year, *n* (%)^b^								
2012	0 (0)	128 645 (13.8)	16 447 (17.1)	30 580 (13.3)	24 810 (12.0)	49 357 (12.7)	249 839 (13.5)	249 839 (12.8)
2013	0 (0)	127 156 (13.6)	15 744 (16.4)	32 588 (14.2)	25 994 (12.5)	46 466 (12.0)	247 948 (13.4)	247 948 (12.7)
2014	0 (0)	131 352 (14.1)	15 589 (16.3)	30 738 (13.4)	27 415 (13.2)	47 153 (12.1)	252 247 (13.6)	252 247 (12.9)
2015	6497 (6.3)	138 766 (14.9)	14 456 (15.1)	32 797 (14.3)	31 402 (15.2)	55 265 (14.2)	272 686 (14.7)	279 183 (14.3)
2016	22 395 (21.8)	136 186 (14.6)	12 047 (12.6)	31 481 (13.7)	30 593 (14.8)	62 694 (16.1)	273 001 (14.7)	295 396 (15.1)
2017	30 846 (30.1)	135 089 (14.5)	11 678 (12.2)	34 645 (15.1)	31 672 (15.3)	61 902 (15.9)	274 986 (14.8)	305 832 (15.6)
2018	42 793 (41.7)	134 396 (14.4)	9969 (10.4)	36 257 (15.8)	35 287 (17.0)	65 397 (16.8)	281 306 (15.2)	324 099 (16.6)

CCI = Charlson comorbidity index, SD = standard deviation.

a The *p* values compared mirabegron with all antimuscarinics and were reported as *p* < 0.0001

b No mirabegron users were included from 2012 to 2014, as mirabegron was reimbursed from 2015 in Taiwan.

### 3.2 Persistence

**Fig. 1:**
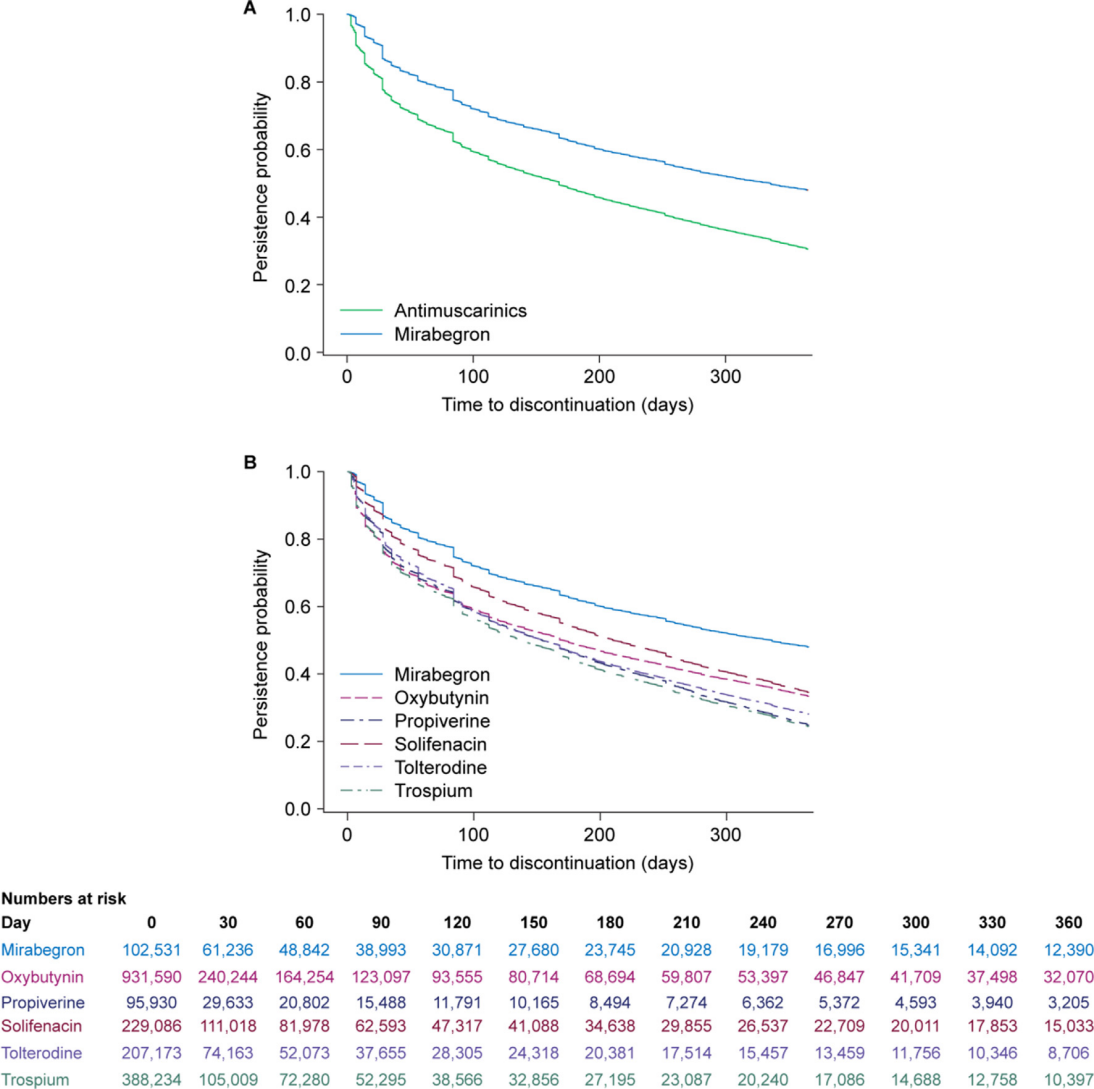
Median time to discontinuation for mirabegron *versus* antimuscarinics (combined [A] and individually [B]). In Panel A, colors represent treatment groups: green = antimuscarinics; blue = mirabegron. In Panel B, colors represent treatment groups: blue = mirabegron; pink = oxybutynin; purple = propiverine; dark red = solifenacin; light purple = tolterodine; green = trospium.

Median (IQR) TTD was 28 (14–54) days overall and was longer with mirabegron (56 [19–168] days) versus all antimuscarinics (14 [5–42] d; Table 2). Concurrently, a difference in slope was noted between mirabegron and antimuscarinics in the Kaplan-Meier curves (Fig. 1). Among the antimuscarinics, TTD was shortest with oxybutynin (7 [3–31] d) and longest with solifenacin (28 [14–98] d). A higher proportion of patients prescribed mirabegron were persistent at 12 mo (11.6%) versus antimuscarinics (3.6%). For antimuscarinics, the persistence rate was lowest with trospium (2.5%) and highest with solifenacin (6.2%). Patients prescribed mirabegron were significantly less likely to discontinue treatment than those receiving antimuscarinics (aHR 1.58 [95% CI 1.55–1.61]; *p* < 0.001).

**Table 2:** Medication persistence and adherence with index drugs (median time to treatment discontinuation [TTD] and median proportion of days covered [PDC])

Parameter	Mirabegron (*n* = 102 531)	Oxybutynin (*n* = 931 590)	Propiverine (*n* = 95 930)	Solifenacin (*n* = 229 086)	Tolterodine (*n* = 207 173)	Trospium (*n* = 388 234)	All antimuscarinics (*n* = 1 852 013)
Medication persistence							
TTD in days, median (IQR)	56 (19–168)	7 (3–31)	14 (7–45)	28 (14–98)	14 (7–60)	7 (3–35)	14 (5–42)^a^
12-mo persistence, % (95% CI)	11.6 (11.4–11.8)	3.3 (3.2–3.3)	3.2 (3.0–3.3)	6.2 (6.1–6.3)	4.0 (3.9–4.1)	2.5 (2.5–2.6)	3.6 (3.5–3.6)
Discontinuation rate							
aHR (95% CI)	Reference	–	–	–	–	–	1.58(1.55–1.61)^a^
Medication adherence							
PDC, median (IQR)	0.18(0.06–0.52)	0.02(0.01–0.08)	0.04(0.02–0.10)	0.08(0.04–0.27)	0.04(0.02–0.15)	0.02(0.01–0.08)	0.03(0.01–0.12)^a^

aHR = adjusted hazard ratio, CI = confidence interval, IQR = interquartile range, PDC = proportion of days covered, TTD = time to discontinuation.

a *p* < 0.001 *versus* mirabegron.

For both types of treatment, median TTD was longer in older (≥60 yr) than in younger (<60 yr) patients (Supplementary Table 1), and individuals receiving mirabegron had a significantly lower risk of discontinuation than those receiving antimuscarinics in all age subgroups Supplementary Table 2. Median TTD was longer in male than in female patients across all drugs, and in treatment-experienced than in treatment-naïve patients prescribed antimuscarinics (Supplementary Table 3). Patients receiving mirabegron had a significantly lower risk (*p* < 0.001) of discontinuation than those receiving antimuscarinics in subgroups of male (aHR [95% CI]: 1.40 [1.37–1.44]), female (1.78 [1.73–1.83]), treatment-experienced (1.64 [1.60–1.68]), and treatment-naïve (1.58 [1.56–1.61]) patients. Changing the period without prescription renewal used to define discontinuation to 15, 60, or 90 d had little impact on persistence compared with the 30 d base case (Supplementary Table 4).

### 3.3 Adherence

**Fig. 2:**
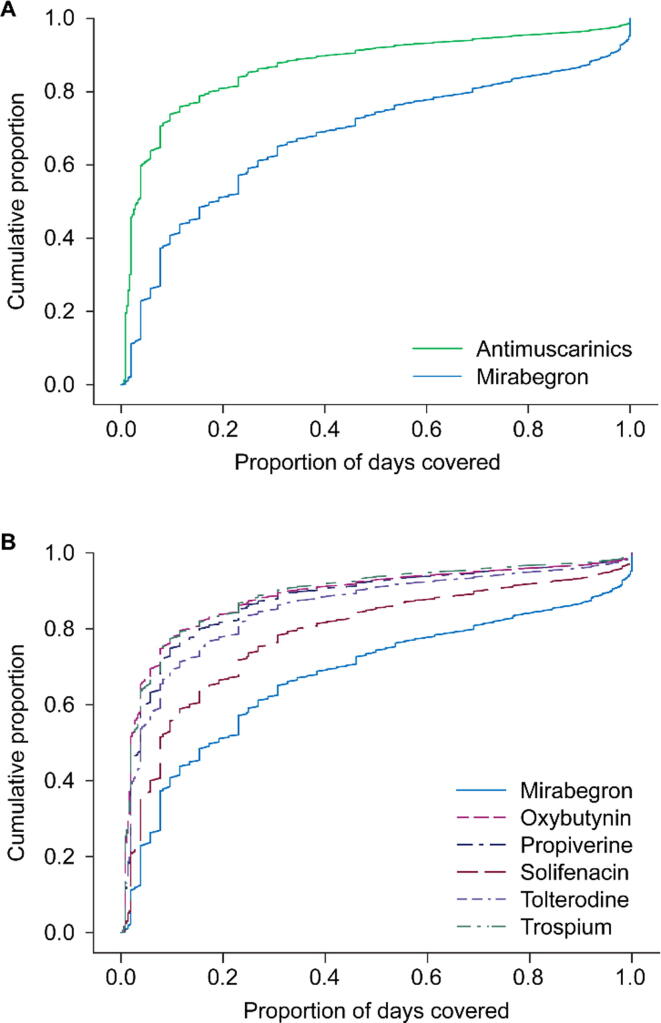
Time to end-of-medication adherence for mirabegron *versus* antimuscarinics (combined [A] and individually [B]). In Panel A, colors represent treatment groups: green = antimuscarinics; blue = mirabegron. In Panel B, colors represent treatment groups: blue = mirabegron; pink dashed = oxybutynin; purple dashed = propiverine; dark red dashed = solifenacin; light purple dashed = tolterodine; green dash-dot = trospium.

Median (IQR) adherence, measured by PDC, was significantly higher with mirabegron (0.18 [0.06–0.52]) than with antimuscarinics (0.03 [0.01–0.12]; *p* < 0.001) (Table 2 and Fig. 2). Similar data were reported for mirabegron *versus* the individual antimuscarinics (PDC ranged from 0.02 with oxybutynin and trospium to 0.08 with solifenacin).

### 3.4 Falls and fractures

PSM was used to improve comparability between groups. After PSM, the distribution of propensity scores was similar between groups (0.927 ± 0.039 vs. 0.927 ± 0.039, *p* = 1.000); however, we were unable to formally assess the balance of individual covariates (eg, standardized mean differences). During a 1-yr follow-up, there were 526 and 358 composite events of falls/fractures among patients receiving mirabegron (23 falls and 503 fractures) and antimuscarinics (12 falls and 346 fractures), respectively. This corresponded to an incidence rates of 0.019 and 0.021 per person-year in the mirabegron and antimuscarinics groups, respectively (Table 3). The adjusted hazards of composite falls/fractures (aHR 1.05 [95% CI 0.91–1.20]; *p* = 0.52), falls (1.33 [0.66–2.68]; *p* = 0.431), and fractures (1.04 [0.90–1.19]; *p* = 0.623) were not significantly different between individuals prescribed mirabegron or antimuscarinics.

**Table 3:** Crude incidence rate and risk of fracture and/or falls with mirabegron and antimuscarinics (propensity-matched COX regression analysis)

Parameter	Mirabegron (*n* = 95 408)	Antimuscarinics (*n* = 95 408)
Composite of falls and fractures		
Events, *n*	526	358
Follow-up PYs	27 167	16 890
IR^a^	0.019	0.021
aHR (95% CI)	Reference	1.05 (0.91–1.20)
*p* value	–	0.519
Fractures		
Events, *n*	503	346
Follow-up PYs	27 180	16 898
IR^a^	0.095	0.021
aHR (95% CI)	Reference	1.04 (0.90–1.19)
*p* value	–	0.623
Falls		
Events, *n*	23	12
Follow-up PYs	27 347	16 984
IR^a^	0.001	0.001
aHR (95% CI)	Reference	1.33 (0.66–2.68)
*p* value	–	0.431

aHR = adjusted hazard ratio, CI = confidence interval, IR = incidence rate, PY = person-yr.

a IRs are rounded to three decimal places; values <0.001 are presented as “<0.001”.

Age subgroup analyses indicated that the incidence of falls/fractures was lower in younger (<70 yr) than in older (≥70 yr) patients (Supplementary Table 5). An association was observed between treatment groups and the incidence of composite falls/fractures when defined as a hospitalization or one outpatient record with corresponding x-ray test; patients prescribed mirabegron had a higher adjusted hazard than those prescribed antimuscarinics (aHR 1.18 [95% CI 1.01–1.38], *p* = 0.038; Supplementary Table 6). Given the large number of comparisons, this isolated finding should be interpreted with caution, as it may reflect chance rather than a true association. All other sensitivity analyses showed a numerically higher incidence of composite falls/fractures with antimuscarinics than with mirabegron.

## 4 Discussion

Real-world data on treatment persistence and adherence with mirabegron and antimuscarinics are limited in populations from East Asia, as are data on the association with falls/fractures. In this study, treatment persistence was significantly longer, and adherence rates were significantly higher, with mirabegron than with antimuscarinics in patients receiving OAB treatment in Taiwan.

The study data showed that greater persistence was also observed with mirabegron (56 d) than with each of the individual antimuscarinics (range 7–28 d). The observed differences in persistence with mirabegron versus antimuscarinics were consistent across subgroups defined by age, sex, and prior treatment. Our study aligns with several retrospective observational studies conducted in other countries [11], [12], [22], including studies in older adults with OAB who are frail [15]. Furthermore, a systematic literature review of oral pharmacotherapy for OAB treatment in a real-world setting reported that persistence and adherence in the overall study populations were longer with mirabegron than with antimuscarinics [23].

The occurrence of drug AEs is a common reason for discontinuation of OAB treatment [24]. A lower incidence of bothersome anticholinergic AEs, such as dry mouth and constipation, observed with mirabegron than with antimuscarinics [7], [8] would be expected to contribute to the observed differences in persistence and adherence between the two drug classes. Other patient-reported reasons for discontinuing OAB treatment (irrespective of class) include no improvement in symptoms, coping without medication, cost, and being told to stop treatment by a health care professional [24], [25]. Additionally, patients with a greater number of comorbidities were observed to have a higher risk for the presence and severity of OAB symptoms [26], [27]. Among these, patients at high risk of cardiovascular disease were reported to experience refractory OAB and derive less benefit from anticholinergic therapy [28], [29]. It is plausible that such patients may discontinue treatment earlier, resulting in shorter persistence with antimuscarinic medications.

Subgroup analyses from our study indicated longer median TTD in older than in younger patients and in male than in female patients in both the mirabegron and antimuscarinic cohorts. Longer persistence with mirabegron and antimuscarinics in older patients has been observed in multiple other real-world studies [12], [21], [25]. One possible explanation is that older patients often have longer disease duration and greater familiarity with OAB therapies, which could influence persistence. Real-world data from Korea also reported improved persistence in men [11], whereas other studies reported greater persistence in women [10], [25].

Patients who received combination treatment (add-on of a second OAB drug) were censored in this analysis, but it is possible that some patients received combination treatment that included an α1-blocker (eg, tamsulosin). Men with lower urinary tract symptoms commonly experience storage symptoms, but α1-blocker monotherapy is often insufficient [30], [31]. Combination therapy of an α1–blocker plus an antimuscarinic or mirabegron is an appropriate treatment strategy for patients with persistent storage symptoms [32], based on improved efficacy and HRQoL in several randomized studies [30], [31]. Among patients receiving mirabegron in this study, more male patients (58.9%) were observed than female patients. This suggests that mirabegron is considered an add-on treatment for male patients, particularly those with minimal risk of urinary retention.

Older individuals with OAB often have higher levels of comorbidity and polypharmacy, receive multiple drugs with anticholinergic activity, and may be more vulnerable to off-target side effects [13]. Several studies suggest that higher rates of falls/fractures are observed in patients with OAB [16], [33] and in those with higher levels of anticholinergic burden [16]. In addition, data suggest that exposure to anticholinergic drugs, including bladder antimuscarinics, is associated with an increased risk of cognitive impairment/dementia [34], [35].

In our study, most falls/fractures were observed in older patients (aged 70–90 yr), although no significant differences were observed between treatments. These findings should be interpreted with caution. Firstly, some risk factors (e.g., frailty, functional status) were not available, and confounding by indication is possible if clinicians preferentially prescribe mirabegron to frailer patients. Secondly, the matched cohorts also had different person-years of follow-up (27 167 vs. 16 890 person-years), reflecting substantial differences in exposure patterns between groups. Thirdly, given that the analysis was based on the index drug and patients receiving antimuscarinics had a relatively short time (median of 14 d), this limited exposure would attenuate differences from the overall anticholinergic burden/exposure, and off-treatment time likely biased HRs toward the null. Furthermore, given this short period of exposure, the total anticholinergic burden could have been similar between the treatment groups. These factors help contextualize the lack of significant difference in falls/fractures between the drug classes in our study. Limited evidence suggests that mirabegron is associated with a lower incidence of falls/fractures than oxybutynin, but not other antimuscarinics (tolterodine, solifenacin, darifenacin, fesoterodine, trospium) [36].

The strengths of this study include a large population of nearly two million patients receiving OAB treatment from the NHIRD, which includes virtually the entire population of Taiwan [20], and data capture from routine clinical practice over a seven yr period. Other strengths include that all the OAB drugs are reimbursed in Taiwan, allowing objective drug selection; the use of PSM to reduce imbalances in baseline characteristics when assessing fall/fracture outcomes; and the corroboration of fall/fracture data using recorded ICD diagnosis codes. There are several limitations in our study. Firstly, treatment persistence and adherence data were based on outpatient visits with a prescription record, and the reasons for discontinuation or switching of OAB treatment were not captured due to the absence of patient-reported medication-taking behavior. Secondly, stepwise Cox regression models were used, which can be sensitive to variable inclusion. Thirdly, due to the retrospective nature of the study, the indication for therapy could not be fully verified, and eligible patients may have received mirabegron or an antimuscarinic for non-OAB symptoms. Fourthly, we could not perform formal covariate balance diagnostics such as standardized mean differences after PSM; thus, we cannot quantify how well observed confounding was controlled, and residual imbalance may persist. Lastly, fewer patients (5.3%) were prescribed mirabegron in the overall study population, which limits the robustness of comparisons between mirabegron and antimuscarinics. However, the use of the largest population-based claims database in Taiwan enabled the inclusion of a comprehensive sample of patients receiving OAB treatment, thereby enhancing the generalizability of our findings.

## 5 Conclusions

Persistence and adherence rates were significantly greater with mirabegron than with antimuscarinics in patients receiving OAB treatment in Taiwan, and persistence was longer in older than in younger patients and in male than in female patients. In contrast to the persistence and adherence data, no significant association was observed between mirabegron and antimuscarinics and the incidence of falls/fractures, although a higher incidence was observed in older than in younger patients. It is noted that the associations are compatible with both potential moderate harm and moderate benefit, and therefore do not provide proven evidence of equivalence regarding falls/fractures. We believe this study may help physicians’ understanding of treatment patterns for OAB pharmacotherapy, including how to effectively utilize mirabegron and antimuscarinics in clinical practice.

  ***Author contributions*:** Chung-Yu Chen had full access to all the data in the study and takes responsibility for the integrity of the data and the accuracy of the data analysis.

  *Study concept and design:* S.H. Chen, C-Y. Chen, Hadi.

*Acquisition of data:* S.H. Chen, C-Y. Chen.

*Analysis and interpretation of data*: S.H. Chen, C-Y. Chen.

*Drafting of the manuscript*: S.H. Chen, C-Y. Chen, Hadi.

*Critical revision of the manuscript for important intellectual content:* S.H. Chen, C-Y. Chen, Hadi.

*Statistical analysis*: S.H. Chen, C-Y. Chen.

*Obtaining funding:* C-Y. Chen.

*Administrative, technical, or material support*: S.H. Chen, C-Y. Chen.

*Supervision:* C-Y. Chen, Hadi.

*Other*: None.

  ***Financial disclosures:*** Chung-Yu Chen certifies that all conflicts of interest, including specific financial interests and relationships and affiliations relevant to the subject matter or materials discussed in the manuscript (eg, employment/affiliation, grants or funding, consultancies, honoraria, stock ownership or options, expert testimony, royalties, or patents filed, received, or pending), are the following: Farid Abdul Hadi is an employee of Astellas Pharma Inc.

  ***Funding/Support and role of the sponsor*:** This study was funded by Astellas Pharma Inc. This work was supported by a grant from the Kaohsiung Medical University (KMU- S114008).

  ***Ethics statement:*** This study was approved by the IRB of Kaohsiung Medical University Hospital (KMUHIRB-E(I)-20210151) and conducted per the Declaration of Helsinki.

  ***Data sharing statement*:** While analytical datasets are maintained within the respective institutions of study, investigators are limited to use within the academic institutions having the necessary privacy policies in place.
