# Supplementary material for: Treatment Persistence and Adherence with Overactive Bladder Medications in Taiwan: A Retrospective Database Analysis
Source: Eur Urol Open Sci. 2026 Apr 21;88:22–30. doi: 10.1016/j.euros.2026.03.020 (PMC13123392; doi:10.1016/j.euros.2026.03.020)
Supplement: Supplementary Data 1 [file mmc1.docx]

**Supplementary materials**

Supplementary Table 1. Medication persistence with index drugs stratified by age (median time to treatment discontinuation [TTD]

Supplementary Table 2. Risk of discontinuation for mirabegron *versus* antimuscarinics in age subgroups (Cox regression analysis)

Supplementary Table 3. Medication persistence with index drugs stratified by sex and treatment status (median time to treatment discontinuation [TTD])

Supplementary Table 4. Sensitivity analysis of medication persistence with index drugs using different periods without prescription renewal (median time to treatment discontinuation [TTD] and 12-month persistence rate)

Supplementary Table 5. Crude incidence rate and risk and association between treatment type and incidence of fracture and/or falls, stratified by age group (propensity-matched COX regression analysis)

Supplementary Table 6. Sensitivity analyses of crude incidence rate and association between treatment type and incidence of fracture and/or falls using different definitions (propensity-matched COX regression analysis)

Supplementary Figure. 1. Patient selection.

Supplementary Figure. 2. Patient selection flowchart.

**Supplementary Table 1. Medication persistence with index drugs stratified by age (median time to treatment discontinuation [TTD])**

| OAB drug | Age in years | | | | | | | | | | | |
| --- | --- | --- | --- | --- | --- | --- | --- | --- | --- | --- | --- | --- |
|  | <50 | | 50 to <60 | | 60 to <70 | | 70 to <80 | | 80 to <90 | | ≥90 | |
|  | N | TTD in days, median (IQR) | N | TTD in days, median (IQR) | N | TTD in days, median (IQR) | N | TTD in days, median (IQR) | N | TTD in days, median (IQR) | N | TTD in days, median (IQR) |
| Mirabegron | 13,638 | 28 (10–60) | 15,796 | 35 (14–111) | 26,739 | 62 (21–175) | 26,576 | 84 (28–217) | 17,016 | 84 (28–245) | 2,766 | 84 (28–237) |
| Oxybutynin | 316,621 | 5 (3–9) | 178,573 | 7 (3–28) | 183,363 | 14 (5–56) | 152,170 | 22 (7–84) | 88,931 | 28 (7–96) | 11,932 | 28 (7–96) |
| Propiverine | 25,028 | 7 (5–16) | 18,478 | 14 (7–35) | 21,017 | 21 (7–84) | 18,919 | 30 (14–105) | 10,910 | 28 (10–130) | 1,578 | 30 (7–84) |
| Solifenacin | 48,019 | 14 (7–35) | 42,244 | 28 (12–84) | 53,149 | 35 (14–112) | 51,222 | 52 (16–147) | 30,379 | 56 (21–168) | 4,073 | 56 (21–172) |
| Tolterodine | 59,637 | 7 (4–14) | 37,529 | 14 (7–44) | 42,818 | 28 (7–84) | 39,314 | 30 (10–97) | 24,597 | 33 (13–108) | 3,548 | 35 (14–113) |
| Trospium | 137,083 | 5 (3–14) | 72,913 | 7 (3–28) | 75,548 | 14 (6–56) | 61,943 | 28 (7–84) | 35,872 | 28 (7–91) | 4,875 | 28 (7–98) |
| All antimuscarinics | 586,118 | 7 (3–14) | 349,737 | 10 (5–33) | 375,895 | 18 (7–73) | 323,568 | 28 (7–91) | 190,689 | 28 (7–111) | 26,006 | 28 (8–112) |

IQR: interquartile range, OAB: overactive bladder, TTD: time to discontinuation.

**Supplementary Table 2. Risk of discontinuation for mirabegron *versus* antimuscarinics in age subgroups (Cox regression analysis)**

|  | aHR (95% CI)^a^ |
| --- | --- |
| Mirabegron | Reference^b^ |
| Age: <50 years | 1.98 (1.88–2.09) |
| Age: 50 to <60 years | 1.70 (1.62–1.78) |
| Age: 60 to <70 years | 1.56 (1.51–1.62) |
| Age: 70 to <80 years | 1.46 (1.40–1.51) |
| Age: 80 to <90 years | 1.40 (1.34–1.47) |
| Age: ≥90 years | 1.33 (1.18–1.50) |

aHR: adjusted hazard ratio, CI: confidence interval.

^a^aHR was calculated using a multivariate Cox regression model, with P-values reported as <0.0001 across all age subgroups

^b^Reference group was applied separately for each of the age groups.

**Supplementary Table 3. Medication persistence with index drugs stratified by sex and treatment status (median time to treatment discontinuation [TTD])**

| OAB drug | Sex | | | | Treatment status | | | |
| --- | --- | --- | --- | --- | --- | --- | --- | --- |
|  | Male | | Female | | Experienced | | Naïve | |
|  | N | TTD in days, median (IQR) | N | TTD in days, median (IQR) | N | TTD in days, median (IQR) | N | TTD in days, median (IQR) |
| Mirabegron | 60,429 | 84 (28–236) | 42,102 | 28 (14–94) | 22,548 | 46 (18–142) | 79,983 | 56 (19–168) |
| Oxybutynin | 347,804 | 28 (7–90) | 583,786 | 7 (3–14) | 39,168 | 20 (7–63) | 892,422 | 7 (3–30) |
| Propiverine | 43,335 | 42 (14–140) | 52,595 | 7 (7–28) | 11,754 | 28 (9–90) | 84,176 | 14 (7–84) |
| Solifenacin | 109,134 | 56 (19–154) | 119,952 | 21 (7–63) | 40,670 | 29 (14–98) | 188,416 | 28 (14–98) |
| Tolterodine | 83,740 | 35 (14–109) | 123,433 | 7 (5–28) | 25,999 | 23 (7–70) | 181,174 | 14 (7–58) |
| Trospium | 166,246 | 15 (4–82) | 221,988 | 7 (3–22) | 37,158 | 28 (7–84) | 351,076 | 7 (3–30) |
| All antimuscarinics | 750,259 | 28 (7–93) | 1,101,754 | 7 (3–22) | 154,749 | 28 (7–84) | 1,697,264 | 10 (4–40) |

IQR: interquartile range, OAB: overactive bladder, TTD: time to discontinuation.

**Supplementary Table 4. Sensitivity analysis of medication persistence with index drugs using different periods without prescription renewal (median time to treatment discontinuation [TTD] and 12-month persistence rate)**

| Parameter  Period without prescription renewal | Mirabegron (n=102,531) | Oxybutynin (n=931,590) | Propiverine (n=95,930) | Solifenacin (n=229,086) | Tolterodine (n=207,173) | Trospium (n=388,234) |
| --- | --- | --- | --- | --- | --- | --- |
| TTD, median (IQR) |  |  |  |  |  |  |
| 30 days^a^ | 56 (19–168) | 7 (3–31) | 14 (7–45) | 28 (14–98) | 14 (7–60) | 7 (3–35) |
| 15 days | 49 (15–153) | 7 (3–28) | 14 (7–42) | 28 (14–91) | 14 (7–56) | 7 (3–30) |
| 60 days | 56 (21–180) | 7 (4–37) | 14 (7–56) | 29 (14–108) | 14 (7–70) | 9 (3–42) |
| 90 days | 63 (21–195) | 8 (4–42) | 14 (7–56) | 30 (14–112) | 15 (7–79) | 10 (3–47) |
| 12-month persistence, % (95% CI) | |  |  |  |  |  |
| 30 days^a^ | 11.6 (11.4–11.8) | 3.3 (3.2–3.3) | 3.1 (3.0–3.3) | 6.2 (6.1–6.3) | 4.0 (3.9–4.1) | 2.5 (2.5–2.6) |
| 15 days | 10.4 (10.2–10.6) | 2.8 (2.8–2.8) | 2.7 (2.6–2.8) | 5.4 (5.3–5.5) | 3.3 (3.3–3.4) | 2.2 (2.1–2.2) |
| 60 days | 12.8 (12.6–13.0) | 3.8 (3.8–3.8) | 3.6 (3.5–3.7) | 7.0 (6.9–7.1) | 4.6 (4.5–4.7) | 2.9 (2.8–2.9) |
| 90 days | 13.6 (13.4–13.8) | 4.1 (4.1–4.2) | 3.9 (3.8–4.0) | 7.5 (7.4–7.6) | 5.0 (4.9–5.1) | 3.1 (3.1–3.2) |

CI: confidence interval, IQR: interquartile range, TTD: time to discontinuation.

^a^Base case analysis.

**Supplementary Table 5. Crude incidence rate and association between treatment type and incidence of fracture and/or falls with mirabegron and antimuscarinics, stratified by age group (propensity-matched COX regression analysis)**

| Parameter | Age in years | | | | | | | | | | | | | | | | | | | | | |
| --- | --- | --- | --- | --- | --- | --- | --- | --- | --- | --- | --- | --- | --- | --- | --- | --- | --- | --- | --- | --- | --- | --- |
|  | <50 | | 50 to <60 | | | | 60 to <70 | | | | 70 to <80 | | | | 80 to <90 | | | | ≥90 | | | |
|  | MIRA^a^ (n=13,169) | AM (n=13,169) | MIRA^a^ (n=15,139) | | AM (n=15,139) | | MIRA^a^ (n=25,248) | | AM (n=25,248) | | MIRA^a^ (n=24,423) | | AM (n=24,423) | | MIRA^a^ (n=15,208) | | AM (n=15,208) | | MIRA^a^ (n=2,221) | | AM (n=2,221) | |
| Composite of falls and fractures | | | |  | |  | |  | |  | |  | |  | |  | |  | |  | |  |
| IR^b^ | 0.011 | 0.017 | 0.007 | | 0.012 | | 0.009 | | 0.011 | | 0.021 | | 0.019 | | 0.037 | | 0.036 | | 0.057 | | 0.071 | |
| aHR (95% CI) | 1.04 (0.51–2.09) | | 0.75 (0.42–1.36) | | | | 0.92 (0.64–1.34) | | | | 1.13 (0.88–1.44) | | | | 1.15 (0.92–1.44) | | | | 0.86 (0.56–1.32) | | | |
| P-value | 0.921 | | 0.346 | | | | 0.677 | | | | 0.337 | | | | 0.226 | | | | 0.493 | | | |
| Fractures |  |  |  | |  | |  | |  | |  | |  | |  | |  | |  | |  | |
| IR^b^ | 0.010 | 0.016 | 0.006 | | 0.011 | | 0.008 | | 0.010 | | 0.020 | | 0.019 | | 0.036 | | 0.035 | | 0.059 | | 0.069 | |
| aHR (95% CI) | 1.07 (0.52–2.21) | | 0.73 (0.40–1.35) | | | | 0.90 (0.61–1.32) | | | | 1.11 (0.87–1.43) | | | | 1.13 (0.90–1.42) | | | | 0.88 (0.57–1.35) | | | |
| P-value | 0.855 | | 0.317 | | | | 0.584 | | | | 0.401 | | | | 0.280 | | | | 0.554 | | | |
| Falls |  |  |  | |  | |  | |  | |  | |  | |  | |  | |  | |  | |
| IR^b^ | 0.001 | 0.001 | 0.001 | | 0.001 | | 0.001 | | <0.001 | | 0.001 | | 0.001 | | 0.001 | | 0.001 | | 0 | | 0.002 | |
| aHR (95% CI) | 0.67 (0.04–10.72) | | 1.10 (0.10–12.54) | | | | 1.50 (0.29–7.85) | | | | 1.53 (0.46–5.10) | | | | 1.70 (0.43–6.60) | | | | NA | | | |
| P-value | 0.774 | | 0.940 | | | | 0.631 | | | | 0.494 | | | | 0.450 | | | | NA | | | |

aHR: adjusted hazard ratio, AM: antimuscarinic, CI: confidence interval, IR: incidence rate, MIRA: mirabegron, NA: not applicable.

^a^Reference group; ^b^IRs are rounded to three decimal places; values <0.001 are presented as “<0.001”.

**Supplementary Table 6. Sensitivity analyses of crude incidence rate and association between treatment type and incidence of fracture and/or falls using different definitions (propensity-matched COX regression analysis)**

| Parameter | Excluding fracture ≤1 year prior to index date | | Hospitalization or three outpatient visits within 1 month | | Hospitalization or two outpatient visits within 1 month | | Hospitalization or one outpatient visit with corresponding X-ray test | |
| --- | --- | --- | --- | --- | --- | --- | --- | --- |
|  | MIRA^a^ (n=94,079) | AM (n=94,079) | MIRA^a^ (n=94,079) | AM (n=94,079) | MIRA^a^ (n=94,079) | AM (n=94,079) | MIRA^a^ (n=94,079) | AM (n=94,079) |
| Composite of falls and fractures | |  |  |  |  |  |  |  |
| Events, n | 312 | 201 | 161 | 106 | 331 | 227 | 447 | 256 |
| Follow-up PYs | 26,864 | 16,720 | 26,918 | 16,752 | 26,860 | 16,717 | 26,945 | 16,708 |
| IR^b^ | 0.012 | 0.012 | 0.006 | 0.006 | 0.012 | 0.014 | 0.017 | 0.015 |
| aHR (95% CI) | 1.07 (0.89–1.28) | | 1.06 (0.82–1.35) | | 1.02 (0.86–1.21) | | 1.18 (1.01–1.38) | |
| P-value | 0.465 | | 0.672 | | 0.796 | | 0.038 | |
| Fractures |  |  |  |  |  |  |  |  |
| Events, n | 289 | 191 | 146 | 100 | 311 | 218 | 424 | 246 |
| Follow-up PYs | 26,876 | 16,729 | 26,929 | 16,754 | 26,872 | 16,725 | 26,836 | 16,717 |
| IR^b^ | 0.011 | 0.011 | 0.005 | 0.006 | 0.012 | 0.013 | 0.016 | 0.015 |
| aHR (95% CI) | 1.04 (0.87–1.25) | | 1.02 (0.79–1.31) | | 1.01 (0.84–1.20) | | 1.17 (1.00–1.37) | |
| P-value | 0.652 | | 0.899 | | 0.947 | | 0.053 | |
| Falls |  |  |  |  |  |  |  |  |
| Events, n | 23 | 10 | 15 | 6 | 20 | 9 | 23 | 10 |
| Follow-up PYs | 26,964 | 16,775 | 26,967 | 16,771 | 26,965 | 16,775 | 29,533 | 16,774 |
| IR^b^ | 0.001 | 0.001 | 0.001 | <0.001 | 0.001 | 0.001 | 0.001 | 0.001 |
| aHR (95% CI) | 1.59 (0.75–3.36) | | 1.70 (0.65–4.42) | | 1.52 (0.69–3.36) | | 1.41 (0.66–3.01) | |
| P-value | 0.227 | | 0.277 | | 0.302 | | 0.378 | |

aHR: adjusted hazard ratio, AM: antimuscarinic, CI: confidence interval, IR: incidence rate, MIRA: mirabegron, PY: person-year.

^a^Reference group; ^b^IRs are rounded to three decimal places; values <0.001 are presented as “<0.001”.


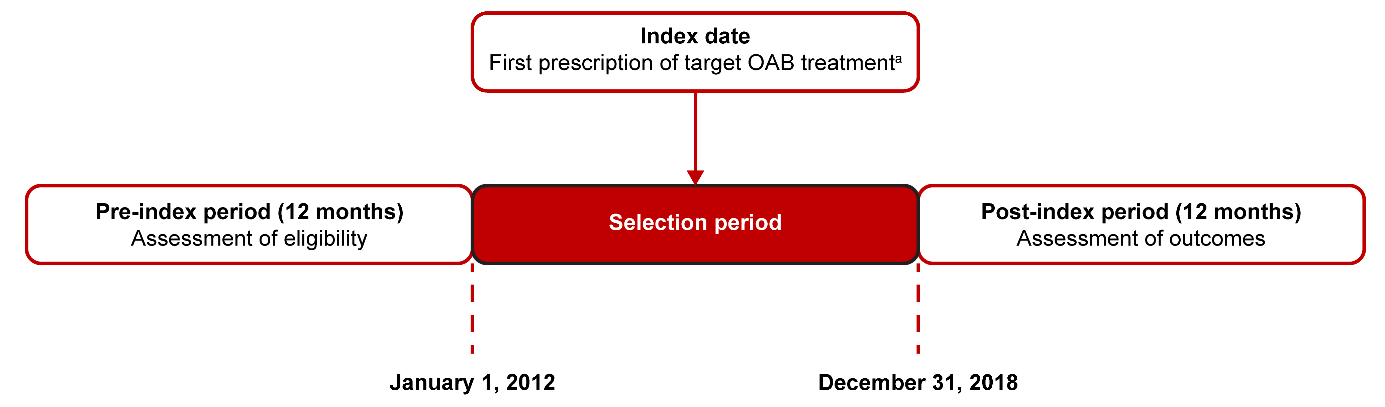


**Supplementary Figure 1. Patient selection.**

^a^Mirabegron, solifenacin, tolterodine, oxybutynin, trospium, or propiverine.

OAB: overactive bladder.

**
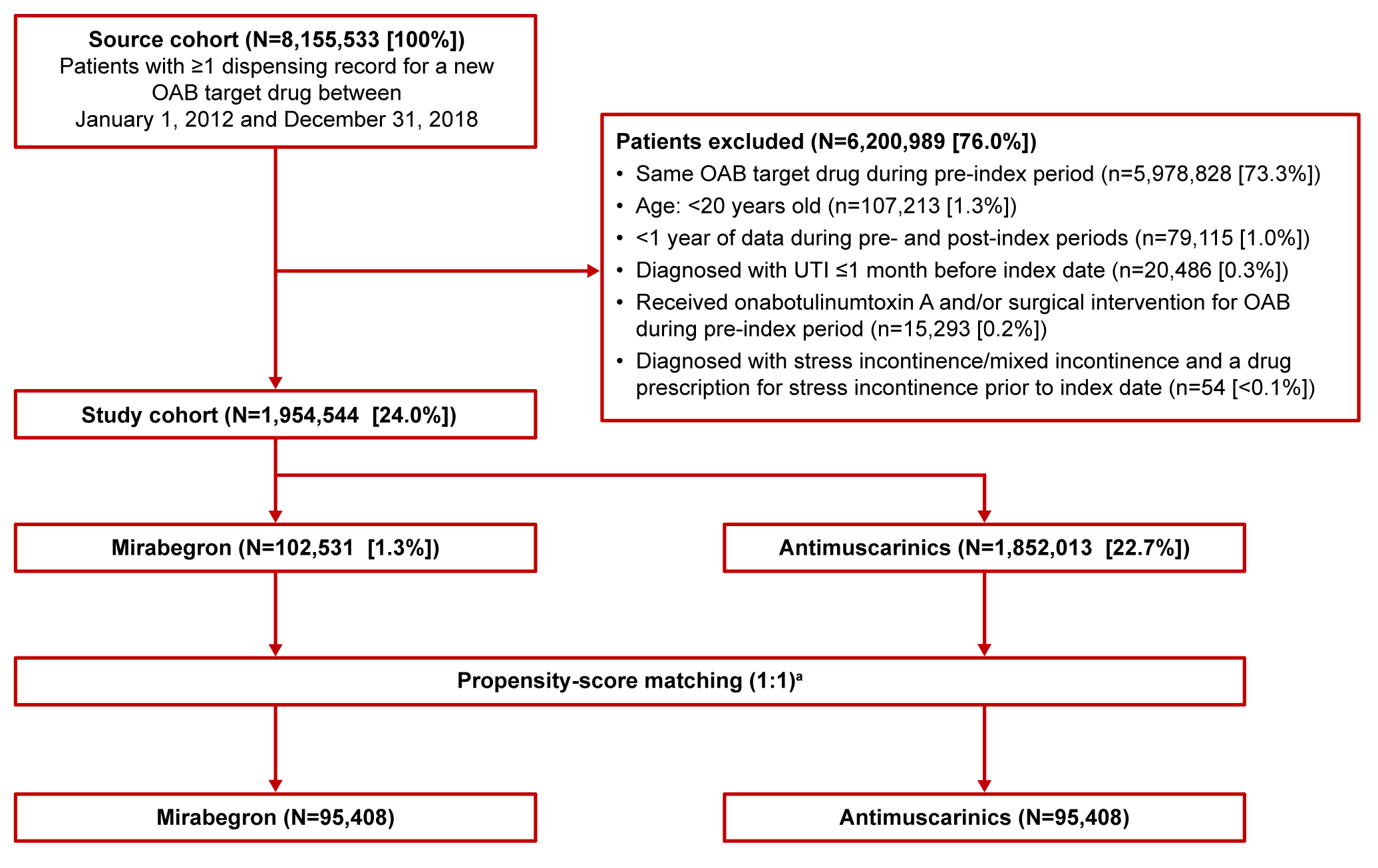
**

**Supplementary Figure. 2. Patient selection flowchart.**
